# Adverse cardiovascular outcomes associated with proton pump inhibitor use after percutaneous coronary intervention: a systematic review and meta-analysis

**DOI:** 10.1186/s12872-024-04029-0

**Published:** 2024-07-17

**Authors:** Bijaya K. Padhi, Mahalaqua Nazli Khatib, Quazi Syed Zahiruddin, Sarvesh Rustagi, Rakesh Kumar Sharma, Ranjit Sah, Prakasini Satapathy, Arathi P. Rao

**Affiliations:** 1 Department of Community Medicine and School of Public Health, Postgraduate Institute of Medical Education and Research, Chandigarh, 160012 India; 2 Division of Evidence Synthesis, Global Consortium of Public Health and Research, Datta Meghe Institute of Higher Education, Wardha, India; 3 South Asia Infant Feeding Research Network (SAIFRN), Division of Evidence Synthesis, Global Consortium of Public Health and Research, Datta Meghe Institute of Higher Education, Wardha, India; 4 School of Applied and Life Sciences, Uttaranchal University, Dehradun, Uttarakhand India; 5 Graphic Era (Deemed to be University), Dehradun, Uttarakhand India; 6 Graphic Era Hill University, Clement Town, Dehradun, India; 7 SR Sanjeevani Hospital, Kalyanpur, Siraha, 56517 Nepal; 8 Department of Clinical Microbiology, Dr. D. Y. Patil Medical College, Hospital and Research Centre, Dr. D. Y. Patil Vidyapeeth, Pune, 411000 Maharashtra India; 9 Center for Global Health Research, Saveetha Medical College and Hospital, Saveetha Institute of Medical and Technical Sciences, Saveetha University, Chennai, 602117 India; 10 Medical Laboratories Techniques Department, AL-Mustaqbal University, Hillah, 51001 Babil Iraq; 11 Dept of Health Policy, Prasanna School of Public Health, Manipal Academy of Higher Education, Manipal, 576104 India; 12 Department of Public Health Dentistry, Dr. D.Y. Patil Dental College and Hospital, Dr. D.Y. Patil Vidyapeeth, Pune 411018, Maharashtra, India

**Keywords:** Proton pump inhibitors, Cardiovascular disease, Myocardial infarction, Percutaneous coronary intervention, Meta-analysis

## Abstract

**Background:**

Proton pump inhibitors (PPIs) are commonly prescribed for gastroprotection in patients undergoing percutaneous coronary intervention (PCI), who are at increased risk of gastrointestinal bleeding due to antiplatelet therapy. However, emerging evidence suggests that PPIs may adversely impact cardiovascular outcomes. This systematic review and meta-analysis sought to assess the relationship between using PPIs and cardiovascular outcomes in patients following PCI.

**Methods:**

We searched various databases up to March 15, 2024, for observational studies and randomized controlled trials (RCTs) assessing the cardiovascular effects of PPIs in PCI patients. Data were extracted on study characteristics, patient demographics, PPI use, and cardiovascular outcomes. The Newcastle-Ottawa Scale and Cochrane Risk of Bias Tool 2 assessed study quality. Meta-analyses were conducted using a random-effects model using R software version 4.3.

**Results:**

A total of 21 studies involving diverse populations and study designs were included. Observational studies suggested a moderate increase in risk for composite cardiovascular diseases (CVD), myocardial infarction (MI), and major adverse cardiac events (MACE) associated with PPI use, with pooled hazard ratios (HRs) of 1.20 (95% CI: 1.093–1.308) for CVD, 1.186 (95% CI: 1.069–1.303) for MI, and 1.155 (95% CI: 1.001–1.309) for MACE. However, RCTs showed no significant link between PPI therapy and negative cardiovascular events (Relative Risk: 1.016, 95% CI: 0.878–1.175). Substantial heterogeneity was observed among observational studies but not RCTs.

**Conclusion:**

The findings indicate that while observational studies suggest a potential risk of adverse cardiovascular events with post-PCI use of PPI, RCTs do not support this association. Further large-scale, high-quality studies are required to understand the cardiovascular implications of individual PPIs better and optimize patient management post-PCI. This analysis shows the complexity of PPI use in patients with coronary artery diseases and the necessity to balance gastroprotective benefits against potential cardiovascular risks.

**Supplementary Information:**

The online version contains supplementary material available at 10.1186/s12872-024-04029-0.

## Introduction

Percutaneous coronary intervention (PCI), commonly referred to as coronary angioplasty, is a minimally invasive technique employed to address obstructive coronary artery disease [1, 2]. It involves the insertion of a catheter into the blocked coronary artery and the subsequent balloon inflation to dilate the artery and improve blood flow. A stent (a small mesh tube) is frequently implanted to keep the artery open. PCI has revolutionized the treatment of coronary artery disease, offering an effective alternative to open-heart surgery for many patients [3].

While PCI has proven to be a life-saving procedure for countless individuals, it is not without potential complications. One significant concern is the increased risk of gastrointestinal (GI) bleeding, particularly among patients who require concomitant antiplatelet therapy, such as aspirin and P2Y12 inhibitors like clopidogrel or prasugrel [3]. While essential for preventing stent thrombosis and reducing the risk of adverse cardiovascular events, these medications can increase the likelihood of GI bleeding by impairing platelet function [4].

To mitigate this risk, proton pump inhibitors (PPIs) are often prescribed prophylactically to patients undergoing PCI. PPIs are a class of medications that potently suppress gastric acid secretion, thereby reducing the risk of peptic ulcers and GI bleeding [5]. Clinical practice guidelines and expert consensus recommendations support their widespread use in this setting. However, studies have raised concerns about potential adverse cardiovascular outcomes linked with PPI use, particularly in individuals with established cardiovascular illnesses [6, 7]. These concerns stem from various proposed mechanisms, including the ability for PPIs to interfere with the antiplatelet effects of clopidogrel, alter endothelial function, promote vascular calcification, and disrupt the gut microbiome, which may have systemic implications for cardiovascular health [8].

Numerous observational studies and meta-analyses have explored the relationship between the use of PPIs and cardiovascular outcomes [6, 8, 9]. However, the results have been inconsistent, often marred by methodological heterogeneity and potential confounding factors. While some studies indicate an elevated risk of adverse cardiovascular events such as myocardial infarction (MI), stroke [10], and cardiovascular mortality associated with PPI use, others report no significant connections or even propose potential protective effects [6, 9]. However, systematic reviews focusing specifically on populations that have undergone PCI are sparse. Additionally, previous meta-analyses have not included many recent studies potentially relevant to this topic.

Given the widespread use of PPIs in patients undergoing PCI and the potential implications for cardiovascular outcomes, it is crucial to thoroughly evaluate the available evidence and provide clarity on this contentious issue. This systematic review and meta-analysis aim to address this critical knowledge gap by rigorously evaluating the current literature on the relationship between PPI use and adverse cardiovascular outcomes in patients who have undergone PCI.

## Methods

A protocol for this study was registered with the International Prospective Register of Systematic Reviews (PROSPERO). The review was performed adhering to the Preferred Reporting Items for Systematic Reviews and Meta-Analyses (PRISMA) guidelines [11] (Table S1).

### Eligibility criteria

We included observational studies cross-sectional, cohort, case-control and randomized controlled trials (RCTs) that assessed the link between PPI use and adverse cardiovascular outcomes in patients who had undergone PCI. Eligibility criteria were established to focus on adult participants (18 years and older) who had undergone PCI, with the intervention of interest being using proton pump inhibitors post-procedure. Comparators included patients not using PPIs post-PCI or those assessed for baseline cardiovascular risk before PPI use. The primary outcomes targeted were myocardial infarction, stroke, cardiovascular mortality, and composite outcomes of these. We limited inclusion to observational studies and RCTS, excluding case reports, editorials, and reviews to ensure the empirical validity of the systematic review.

### Information sources and search strategy

A literature search was conducted in databases including PubMed, EMBASE, and Web of Science, from inception to March 15, 2024, with no language restrictions. The search strategy combined terms related to “proton pump inhibitors,” and “cardiovascular outcomes.” An experienced librarian reviewed the search strategy to ensure comprehensiveness. The full search strategy is given in Table S2.

### Study selection

Titles and abstracts were initially screened for eligibility by two independent reviewers, followed by a detailed full-text evaluation of studies that potentially met the inclusion criteria. Any disagreements among the reviewers were settled by consulting a third reviewer. We used a semi-automated software (Nested-Knowledge, MN, USA) for screening. The selection process was documented and presented in a PRISMA flow diagram.

### Data extraction

A data extraction form was used to collect data from included studies, such as study characteristics, participant demographics, details of PPI use, cardiovascular outcomes, and confounders adjusted for in the analysis. Two reviewers performed data extraction independently, with difference of opinion resolved through discussion or involving a third reviewer. We employed the tagging function of Nested-knowledge for data extraction.

### Risk of Bias Assessment

Appropriate methodologies were employed to assess the quality of the studies incorporated in this analysis. The Newcastle-Ottawa Scale was used for observational studies, and the Cochrane Risk of Bias Tool 2 (RoB-2) was applied to RCTs. Two independent evaluators carried out these assessments. In cases of divergent opinions, resolutions were achieved by either reaching a consensus or involving a third reviewer for additional input.

### Data synthesis and analysis

Meta-analysis used a random-effects model to account for potential heterogeneity across studies. We pooled hazard ratios (HRs) and confidence intervals for cardiovascular outcomes. Relative risks (RRs) were also calculated from RCTs. Heterogeneity was quantified using the I² statistic, and τ² was estimated to assess the variance between studies [12]. Subgroup analyses were conducted based on the type of PPI and specific cardiovascular outcomes. The evaluation of publication bias was performed using funnel plots and Egger’s test. All statistical analyses were carried out using the R statistical software package (version 4.3), employing the “meta” and “metafor” packages for meta-analysis tasks [13].

## Results

### Literature search

**Fig. 1:**
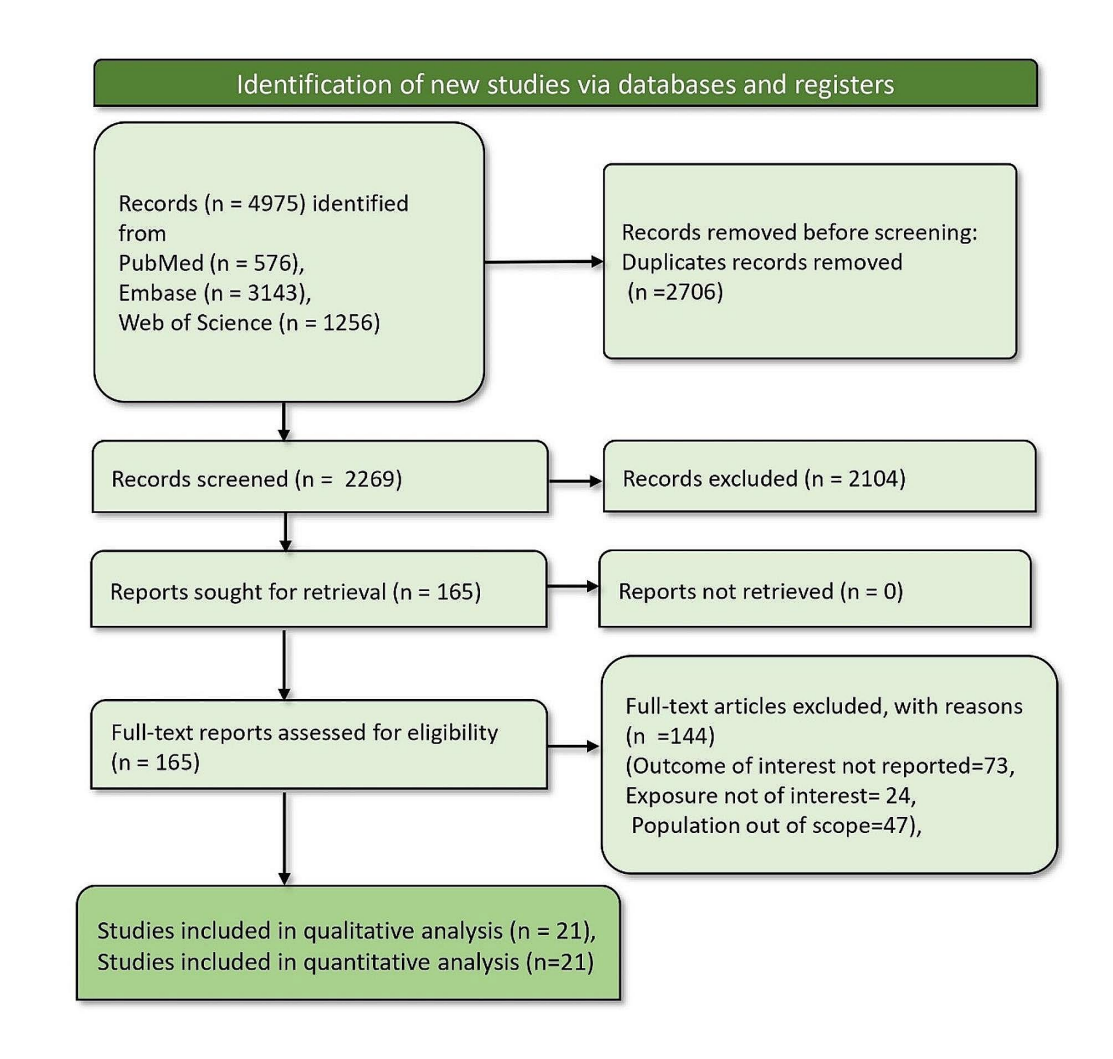
PRISMA flow diagram depicting article screening and selection process

The literature search across various databases yielded a total of 4,975 records. After removing 2,706 duplicate records, 2,269 records were screened. Subsequent evaluation excluded 2,104 records, leaving 165 reports that were sought for retrieval. All 165 full-text reports underwent evaluation to determine their eligibility. Out of these, 144 were excluded due to the following reasons: the outcome of interest was not reported in 73 articles, the exposure was not of interest in 24 articles, and the population was out of scope in 47 articles. Ultimately, 21 studies met the inclusion criteria and were included in qualitative and quantitative analyses [14–34]. Figure 1 depicts the PRISMA flow diagram of the process.

### Characteristics of included studies

The included studies in this systematic review exhibited a range of designs, geographic locations, and outcome measures. Table 1 displays the important characteristics of included studies. They predominantly comprised retrospective cohort studies, RCTs, and a few prospective cohort studies. Geographically, the studies were diverse, originating from multiple countries including China, the Netherlands, Italy, Sweden, Japan, the USA, and others, reflecting a global perspective on the subject. The mean age of participants across the studies ranged from approximately 59 to 71 years, indicating a predominantly older adult population. Male predominance was noted in most studies, with the percentage of male participants varying from around 24.7–82.36%. Sample sizes varied widely, from small-scale studies with as few as 86 participants to large cohorts involving up to 99,836 individuals. Regarding medications, the studies focused on patients who had undergone PCI and were prescribed a range of antiplatelet therapies including aspirin, clopidogrel, ticagrelor, and others. The type of population within these studies included those with acute coronary syndromes (ACS), stable angina, and those undergoing elective or emergent PCI. A variety of PPIs were investigated, including omeprazole, pantoprazole, esomeprazole, and lansoprazole. Follow-up periods also showed variability, ranging from 30 days to up to 3 years, allowing for short-term and long-term outcome assessments. The HRs for cardiovascular outcomes, where reported, were provided with 95% CIs, revealing a range of associations from non-significant to moderately increased risks in observational studies. Adjusted variables in the studies were comprehensive, including factors such as sex, age, body mass index (BMI), diabetes, hypertension, previous MI, and others. However, not all studies provided details on adjusted variables. Risk of bias assessment if given in Table S3 and Table S4.

**Table 1:** Characteristics of included studies

Author	Year	Design	Country	Mean age	Male %	Sample size	Medications	Type of Population	Type of control	HR for CVD outcome (95% CI)	Type of PPIs	Follow up	Adjusted variables
Burkard 2011 [15]	2011	Retrospective analysis of RCT	Netherlands	66.5	78.8	109	Clopidogrel, ASA	Patient who underwent PCI	No PPI users	MI = 1.88 (1.05–3.37)	Esomeprazole, pantoprazole, omeprazole and lansoprazole	3 years	NA
Liu 2022 [21]	2022	Retrospective cohort study	China	62.2	81.2	3027	Aspirin, clopidogrel	STEMI patients undergoing PCI	Non-PPI users	MACE = 2.42 (1.43–4.08)	NA	NA	Age, sex, diabetes, anemia, hypertension, smoking, COPD, prior MI, Prior stroke, femoral access, e-GFR
Macaione 2012 [22]	2012	Retrospective study	Italy	64.31	82.36	176	Aspirin and clopidogrel	ACS patients started after PCI undergoing stent implantation	Anti-H2 group	NA	Omeprazole, Esomeprazole, Lansoprazole, Pantoprazole	3 years	NA
Maret-Ouda 2022 [23]	2022	Retrospective cohort study	Sweden	69	70.8	99,836	Clopidogrel	Patient who underwent PCI	Non-PPI users	MI = 1.23 (1.15–1.32), CHD = 1.28 (1.24–1.33), Stroke = 1.21 (1.05–1.40), Cardiac mortality = 1.52 (1.37–1.69)	Omeprazole, pantoprazole, esomeprazole	1 year	Sex, age, calendar year, obesity-related diseases, tobacco-related diseases, hypertension, CHF, and Charlson comorbidity score
Ono 2022 [25]	2022	Post hoc analysis of RCT	Multiple countries	64.9	24.7	15,839	ASA, clopidogrel/ticagrelor + Aspirin/tricaglator monotherapy	Patients who underwent PCI	Non-PPI users	Any MI = 1.38 (0.99–1.91), Stroke = 2.18 (1.23–3.86)	NA	2 years	Age, sex, BMI, clinical presentation (CCS vs. ACS), diabetes, hypertension, hypercholesterolemia, PVD, COPD, current smoker, renal failure, previous stroke, previous MI, previous PCI, CABG, previous bleeding, PCI for left main CAD, and multivessel disease
Zhang 2020 [32]	2020	RCT	China	60.2	31	86	Aspirin, ticagrelor	Patients with AMI who underwent primary PCI	Placebo	NA	Omeprazole	6 months	NA
Zhu 2017 [33]	2017	Propensity score analysis	China	60.2	75.4	7868	Aspirin, clopidogrel	Patients who underwent PCI	No PPI users	MACCE = 0.970 (0.808–1.165), MI = 0.904 (0.597–1.368), Stroke = 0.730 (0.409–1.302)	NA	1 year	Age, gender, hypertension, dyslipidemia, diabetes mellitus, prior CVA, prior MI, prior PCI, prior CABG, acute MI, ejection fraction, Killip class, e-GFR, hemoglobin, intra-aortic balloon pump, and warfarin use
Zou 2014 [34]	2014	Retrospective cohort study	China	66.2	73.5	7653	Aspirin, clopidogrel	ACS patients started DAT after PCI with DES	No PPI users	ST = 2.66 (1.16–5.87), MACE = 1.33 (1.12–1.5), MI = 1.79 (0.88–3.72)	Omeprazole, pantoprazole, and esomeprazole	1 year	Age, hyperlipidemia, diabetes mellitus, previous MI, unstable angina, GI disease, creatinine, LVEF, ACEI, CCB, and total number of stents
Aihara 2012 [14]	2012	Retrospective Cohort study	Japan	69	72.6	1887	Ethyl icosapentate, Warfarin, Statins, ACE inhibitors, ARBs, β-blockers, Nitrate, Diuretics	Patients who underwent PCI, including coronary stenting	Non-PPI users	MACE = 0.98 (0.53–1.79), MI = 0.69 (0.20–2.32), Coronary revascularization = 1.27 (0.93–1.76), Stroke = 1.21 (0.48–3.19), HF = 1.87 (0.71–5.84)	Lansoprazole, omeprazole, rabeprazole	12 Months	NA
Chandrasekhar 2016 [16]	2016	Cohort study	USA	64.4	73.9	4635	Aspirin, warfarin, Thienopyridine, PPI	Clopidogrel treated PCI Patient	Non-PPI users	MACE = 1.27, 95% CI: 1.04–1.55	Pantoprazole, rabeprazole, omeprazole, esomeprazole, lansoprazole	24 Months	Age, sex, BMI, smoking status, prior MI, prior PCI, education status, center location, stable angina, bivalirudin use, bifurcation lesion, stent type, total stent length, warfarin at discharge and DAPT cessation
Dunn 2013 [17]	2013	Clinical trials (CREDO)	USA	61.8	70.3	2116	NA	CREDO trial patients (Patients undergoing PCI with clopidogrel)	Non-PPI users	Composite outcome = 1.67 (1.06 to 2.64)	Omeprazole, Lansoprazole, Pantoprazole, Rabeprazole	1 year	Race, diabetes, hyperlipidemia, CHF, atrial fibrillation, Different types of previous CVDs, PCI, coronary angiography, and CABG
Gargiulo 2016 [18]	2016	RCT	Italy	71.2	72.5	1970	Aspirin, Clopidogrel, Statin	Patients with PCI and dual-antiplatelet therapy (DAPT) with clopidogrel plus aspirin.	Non-PPI users	MI/CVA = 1.051 (0.788-1.400)	Pantoprazole, omeprazole, esomeprazole, and rabeprazole	2 years	Sex, age, creatinine clearance, clinical presentation, and CRUSADE score
Harjai 2011 [19]	2011	Prospective cohort study	USA	67	62	2651	Aspirin, Warfarin, Statin, β-blockers, ACE-1, ARB, CCB	Patients who underwent PCI	Non-PPI users	MACE = 0.89 (0.63–1.27), MI = 1.04 (0.64–1.69)	Omeprazole, Esomeprazole,	12 months	NA
Sarafoff 2010 [27]	2010	Retrospective cohort study	Germany	66.3	77.5	3,338	NA	Patient who underwent PCI and are on Clopidogrel	Non-PPI users	MI = 1.5 (0.9–2.5)	Pantoprazole, esomeprazole, omeprazole, lansoprazole	30 days	NA
Tentzeris 2010 [28]	2010	Retrospective cohort study	Austria	64.11	65.4	1210	NA	Patients who underwent PCI	Non-PPI users	Cardio vascular death = 0.563 (0.205–1.550), Readmission for ACS = 1.274 (0.285–5.698)	Pantoprazole, esomeprazole, omeprazole, lansoprazole and rabeprazole	7.8 months	NA
Weisz 2015 [30]	2015	Cohort study	USA and Germany.	64.4	70.1	8582	Aspirin, Clopidogrel	Patients who underwent PCI	Non-PPI users	MACE = 1.21 (1.04–1.42), MI = 1.03 (0.78–1.35)	NA	2 years	NA
Nicolau 2020 [24]	2020	RCT	USA	71	74.3	2678	Ticagrelor, warfarin, clopidogrel, dabigatran, Oral hypoglycaemic drugs, ACE inhibitors, ARBs, β-blockers	Patients who underwent PCI	Non-PPI users	Thromboembolic events, Death	CVD death or thromboembolic event = 0.96 (0.66–1.40)	NA	NA
Jensen 2017 [20]	2017	RCT	Denmark	64.7	73.1	2009	NSAID, SSRI, Corticosteroid, Oral anticoagulant	Patients who underwent PCI	Non-PPI users	Unstable angina = 0.752 (2.8-3.0), MI = 0.951 (13.1–13.5)	Pantoprazole	1 year	NA
Ren 2011 [26]	2011	RCT	China	62	72.1	172	Clopidogrel, Aspirin	ACS undergoing elective PCI	Clopidogrel, Aspirin	NA	Omeprazole	1 Month	NA
Wei 2016 [29]	2016	RCT	China	59	56.1	207	Clopidogrel, Aspirin	STEMI undergoing emergent PCI	Clopidogrel, Aspirin	NA	Pantoprazole	6 months	NA
Yano 2012 [31]	2012	RCT	Japan	67	77	130	Clopidogrel, Aspirin, β-blockers, ACE inhibitors, CCB	ACS patient who underwent PCI	Patient of Famotidine	NA	Omeprazole	12 Months	NA

**Abbreviations**: ACS - Acute Coronary Syndrome, ACE-1 - Angiotensin-Converting Enzyme Inhibitors, ARB - Angiotensin II Receptor Blockers, ASA - Aspirin, BMI - Body Mass Index, CABG - Coronary Artery Bypass Grafting, CCS - Chronic Coronary Syndrome, CHD - Coronary Heart Disease, CHF - Congestive Heart Failure, CVA - Cerebrovascular Accident, CVD - Cardiovascular Disease, DES - Drug-Eluting Stent, DAPT - Dual Antiplatelet Therapy, e-GFR - Estimated Glomerular Filtration Rate, GI - Gastrointestinal, HF - Heart Failure, HR - Hazard Ratio, HTN - Hypertension, LVEF - Left Ventricular Ejection Fraction, MACE - Major Adverse Cardiac Events, MACCE - Major Adverse Cardiovascular and Cerebrovascular Events, MI - Myocardial Infarction, NA - Not Available, NSAID - Nonsteroidal Anti-Inflammatory Drug, OR - Odds Ratio, PCI - Percutaneous Coronary Intervention, PPI - Proton Pump Inhibitor, PVD - Peripheral Vascular Disease, RCT - Randomized Controlled Trial, RR - Relative Risk, SSRI - Selective Serotonin Reuptake Inhibitor, STEMI - ST-Elevation Myocardial Infarction

Table 2 presents the clinical characteristics of patients from various studies. Observational studies generally report higher rates of common comorbidities such as hypertension and hyperlipidemia/dyslipidemia, with pooled prevalences of 68.3% and 64.1% respectively. Prevalence of diabetes and smoking was also noted among the patients. A significant observation is the detailed reporting of prior cardiovascular interventions and conditions (such as prior PCI, CABG, MI, PAD, and strokes) in observational studies, unlike in RCTs, where such information may be underrepresented or selectively excluded. There are noticeable differences in the reported rates of conditions, indicating differences in the populations studied.

**Table 2:** Clinical characteristics of patients

Study	Hypertension	Hyperlipidemia/Dyslipidemia	Diabetes	Current smokers	Family history of CVD	Prior PCI	Prior CABG	Prior of MI	Prior PAD	Prior stroke	Indication for PCI			
											STEMI	ACS	Angina pectoris	MI
Aihara 2012	70.1%	83.4%	40.0%	43.9%	10.8	18.4%	5.4%	5%	4.2%	6.3%	NA	NA	NA	NA
Burkard 2011	66.6%	41.8%	19%	29.3%	38.4	16.4%	13.0%	NA	NA	NA	22.0%	36.3%	42.6%	NA
Chandrasekhar 2016	80.3%	75.8%	33%	19.5%	31.4	36.3%	13.8%	24.0%	7.9%	3.6%	NA	40.3%	48.2%	NA
Dunn 2013 (CAPRIE)	51.5%	NA	20.0%	29.5%	NA	NA	NA	NA	NA	NA	NA	NA	NA	32.8%
Dunn 2013 (CREDO)	68.65%	74.4%	26.0%	NA	42.2	27.9%	15.8%	34%	8.3%	6.7%	NA	NA	85.6%	19.9%
Harjai 2011	67.3%	72.6%	28.0%	24.7%	NA	19.3%	17.2%	21.0%	12.6%	NA	NA	NA	NA	NA
Liu 2022	48.0%	12.4%	27.0%	44.2%	NA	7.5%	NA	19%	NA	4.8%	100%	NA	NA	NA
Macaione 2012	73.9%	55.1%	44.%	34.7%	35.8	21.6%	6.2%	16%	NA	NA	NA	100%	NA	NA
Maret-Ouda 2022	55.0%	NA	NA	NA	NA	NA	NA	8%	NA	NA	NA	NA	34.1%	22.6%
Ono 2022	78.6%	NA	25.0%	26.1%	NA	32.8%	5.9%	23%	NA	2.6%	13.0%	46.8%	12.7%	NA
Ren 2011	NA	NA	NA	NA	NA	NA	NA	NA	NA	NA	NA	100%	NA	NA
Sarafoff 2010	65.5%	67.5%	23.0%	17.0%	NA	NA	NA	31%	NA	NA	NA	NA	89.5%	10.5%
Tentzeris 2010	75.6%	76.7%	22.0%	25.9%	NA	14.1%	4.5%	19%	NA	NA	NA	44.5%	NA	NA
Weisz 2015	79.1%	74.4%	32.0%	22.6%	NA	42.8%	17.1%	25%	10.2%	NA	9.5%	NA	NA	NA
Zhu 2017	64.7%	69.3%	30.0%	57.0%	NA	37.1%	5.9%	27%	3.4%	NA	NA	NA	NA	22.0%
Zou 2014	71.1%	60.6%	25.0%	32.0%	NA	NA	NA	18%	NA	NA	NA	NA	NA	NA
Pooled	68.3% (95%: 62.7–73.5%)	64. 1% (95% CI:49.5–76.5%)	28.0% (95% CI:24.0–32.0%)	30.4% (95% CI:24.1–37.4%)	20.9% (95% CI:12.9–31.0%)	23.2% (95%CI:16.3–32.1%)	9.5% (95%CI:6.3–13.69%)	20% (95% CI:5–26%)	7.1% (95% Ci: 4.2–12.0%)	4.5% (95% CI: 2.8–7.3%	60.4% (95%CI: 0.1–100%	81% (95% CI:14.7 to 99.1%)	54.0% (95% CI: 18.9–85.5%)	19.4% ( 95% CI:10.6–32.8%)
RCTs														
Gargiulo 2016	71.8%	54.7%	24.0%	23.8%	NA	17.7%	NA	22%	NA	NA	32.9%	74.4%	44.2%	NA
Jensen 2017	NA	NA	NA	26.5%	NA	NA	NA	NA	NA	NA	NA	NA	NA	NA
Nicolau 2020	51.9%	NA	36.0%	12.5%	NA	33.5%	NA	26%	NA	NA	NA	50.5%	NA	NA
Wei 2016	NA	NA	NA	NA	NA	NA	NA	NA	NA	NA	100%	NA	NA	NA
Yano 2012	65.2%	58.5%	21.0%	57.0%	NA	5.2%	0.7%	3%	NA	NA	53.3%	100%	NA	NA
Zhang 2020	54.7%	72.1%	51.0%	NA	20.9	NA	NA	NA	NA	NA	67.4%	NA	NA	NA
Pooled	61.4% (95% CI: 45.4–75.3%)	NA	NA	29.6% (95% CI:23.6–36.4%)		15.9% (95% CI:1.2–74.5%)	NA	15% (95% CI:0.00–59.0%)	NA	NA	69.9% (95% CI: 10.1–98.0%)	87.4% (95% CI: 0.6–100%	NA	NA

Abbreviations: ACS - Acute Coronary Syndrome, CABG - Coronary Artery Bypass Grafting, CAPRIE - Clopidogrel versus Aspirin in Patients at Risk of Ischaemic Events, CI - Confidence Interval, CVD - Cardiovascular Disease, MI - Myocardial Infarction, NA - Not Available, PAD - Peripheral Arterial Disease, PCI - Percutaneous Coronary Intervention, STEMI - ST-Elevation Myocardial Infarction

### PPI use and CVD outcomes from observational studies

**Fig. 2:**
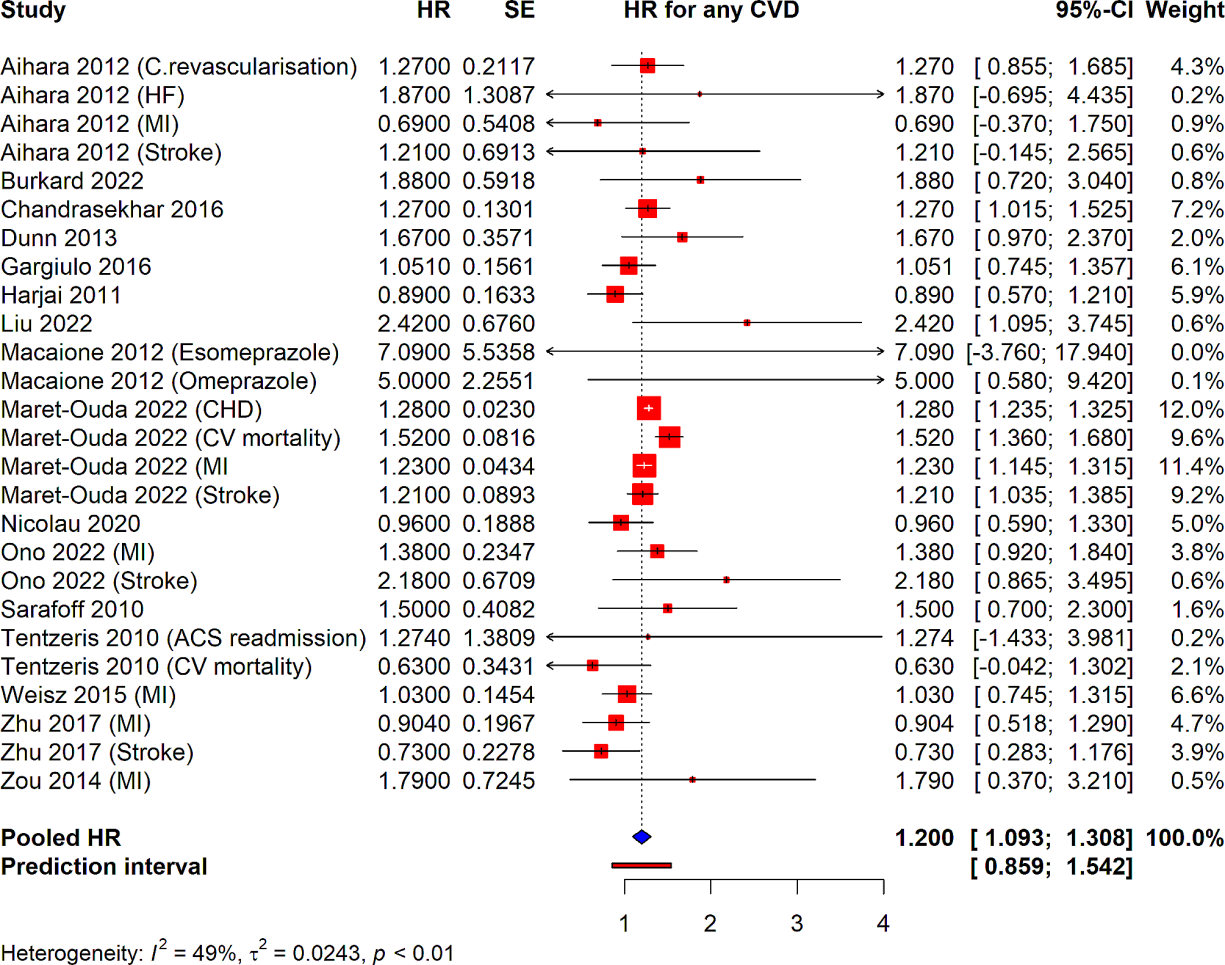
Forest plot depicting association of PPI use and risk of any CVD in patients underwent PCI from observational studies

Observational studies investigating the association between PPI use and CVDs in PCI patients, a diverse array of studies reported on various CVD outcomes. The pooled HR across studies suggested a moderate relationship between PPI use and elevated risk of any CVD outcome, with a pooled HR of 1.20 (95% CI: 1.093–1.308). This indicates that PPI use was associated with an approximately 12% increase in the risk of any cardiovascular event. Individual study HRs varied considerably, with some studies showing a significantly increased risk of specific CVD outcomes, such as coronary revascularization, MI, and stroke, while others reported non-significant associations (Fig. 2). The heterogeneity among studies was moderate, with an I² value of 51%, and the τ² statistic was 0.0206, indicating some variability in the true effect sizes across studies. The *p*-value for heterogeneity was less than 0.01, confirming the presence of statistically significant heterogeneity. The 95% prediction interval was calculated from 0.859 to 1.542.

**Fig. 3:**
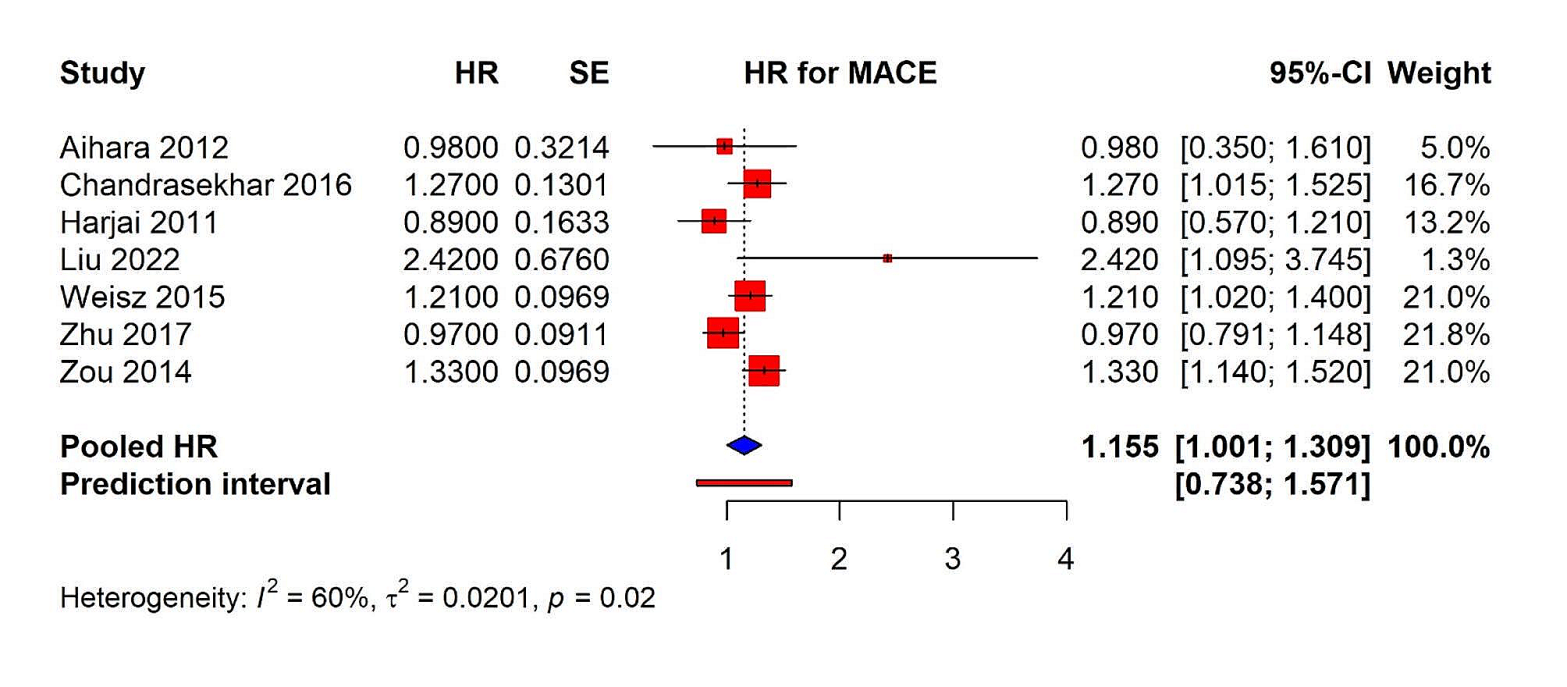
Forest plot depicting association of PPI use and risk of MACE in patients underwent PCI from observational studies

The pooled HR for MACE was calculated at 1.155 (95% CI: 1.001–1.309), signifying a statistically significant increase in the risk of MACE associated with PPI use. This indicates that the use of PPIs was associated with a 15.5% increased risk of major cardiac events, including myocardial infarction, stroke, or cardiovascular death. There was moderate heterogeneity observed across the included studies, with an I² of 60% and a τ² of 0.0201, which suggests variability in the effect estimates that the individual studies reported. The *p*-value for heterogeneity was 0.02, indicating the presence of statistically significant variability among the study outcomes (Fig. 3). The 95% prediction interval was calculated from 0.738 to 1.571.

**Fig. 4:**
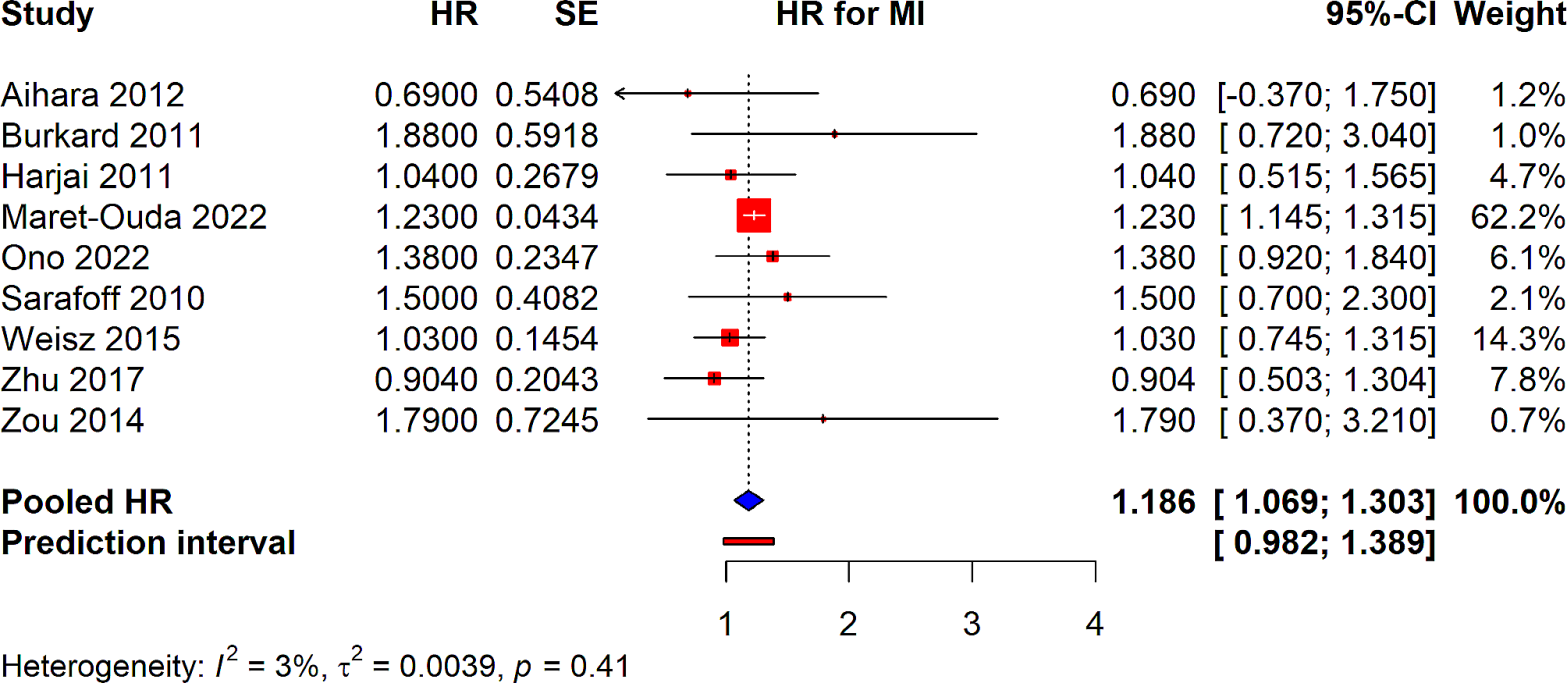
Forest plot depicting association of PPI use and risk of MI in patients underwent PCI from observational studies

The overall pooled HR for MI was determined to be 1.186 (95% CI: 1.069–1.303), indicating an approximately 18.6% increased risk of myocardial infarction associated with the use of PPIs. This finding suggests a significant association between PPI use and the occurrence of MI in the studied populations. Heterogeneity amongst the included studies was minimal, with an I² value of 3% and τ² of 0.0039, suggesting a high level of consistency in the effects reported across studies. The *p*-value for heterogeneity was 0.41, further indicating that there was not significant variation between the studies’ results (Fig. 4). The 95% prediction interval was calculated from 0.982 to 1.389.

**Fig. 5:**
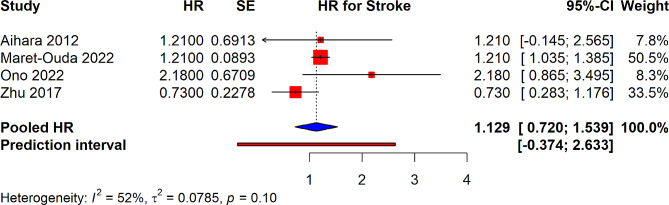
Forest plot depicting association of PPI use and risk of stroke in patients underwent PCI from observational studies

In the analysis of studies examining the risk of stroke linked with PPI use, the pooled HR was found to be 1.129 (95% CI: 0.720–1.539). This suggests a potential 12.9% increase in the risk of stroke among PPI users compared to non-users, although the confidence interval indicates that this increase may not be statistically significant. The heterogeneity among the included studies was moderate with an I² value of 52% (Fig. 5).

### PPI use and CVD outcomes from RCTs

**Fig. 6:**
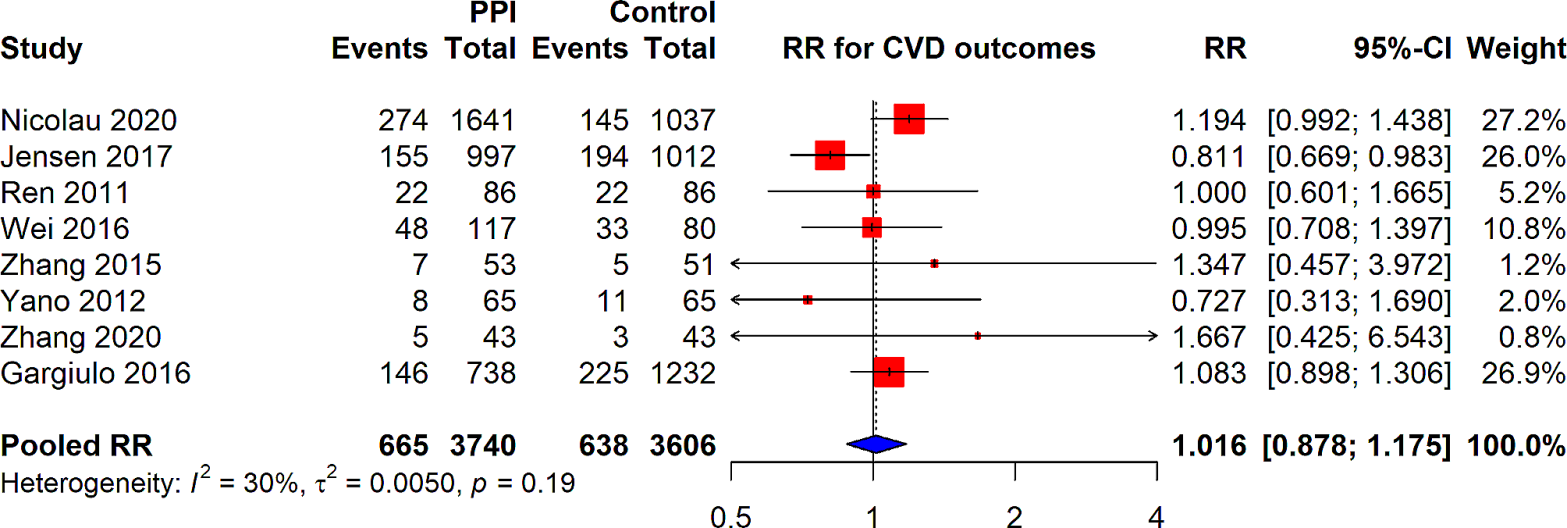
Forest plot depicting association of PPI use and any CVD outcome in patients underwent PCI from RCTs

Six RCTs reported CVD outcome with PPI use in patients undergoing PCI. In this meta-analysis of RCTs assessing the relationship between PPI use and CVD outcomes, the pooled RR was found to be 1.016 (95% CI: 0.878–1.175). This analysis 3,740 individuals in the PPI group and 3,606 individuals in the control group, with a total of 665 and 638 cardiovascular events, respectively. The overall heterogeneity among the studies was low, with an I² of 30%, and a tau-squared (τ²) of 0.0050, indicating relatively little variation between the study outcomes. The *p*-value for heterogeneity was 0.19, suggesting that there is no statistically significant inconsistency across the included studies (Fig. 6).

### Publication bias

We used funnel plot and Egger’s test to evaluate the publications bias (Figures S1 and S2). The analysis revealed no significant publication bias for either observational studies (Egger’s test *p*-value = 0.335) or RCTs (Egger’s test *p*-value = 0.907).

## Discussion

This meta-analysis critically evaluates the relationship between PPI use and adverse cardiovascular outcomes following PCI. Amid conflicting evidence from observational studies and RCTs, our analysis offers a contemporary synthesis, casting light on a controversial topic in cardiovascular pharmacotherapy. While PPI treatment was associated with an elevated risk of adverse cardiovascular outcomes in meta-analyses of observational studies, no such association was observed in patients with PCI in the RCT meta-analysis.

There are several proposed mechanisms by which PPIs may elevate the CVD risk. co-administration of PPIs and dual antiplatelet therapy (DAPT) may elevate cardiovascular risk. Prior research suggests that PPI treatment could reduce aspirin’s absorption and oral bioavailability by inhibiting gastric acid secretion. Some PPIs, including omeprazole and esomeprazole, share a metabolic pathway with clopidogrel, a prodrug, potentially affecting its antiplatelet function through competitive enzymatic interaction. Additional hypotheses suggest that PPIs may elevate plasma levels of asymmetric dimethylarginine, which inhibits nitric oxide synthase, thereby disrupting nitric oxide synthesis. PPIs may also undermine the effectiveness of clopidogrel due to shared cytochrome P450 metabolism, leading to concerns about their concurrent use and potential for cardiovascular harm.

Our meta-analysis of RCTs revealed no significant link between PPI use and cardiovascular outcomes in PCI patients, with minimal study heterogeneity. Conversely, the observational studies meta-analysis showed a significant increase in composite CVD, MACE, and MI risks, but not for stroke. The variability in outcomes might be ascribed to differences in study designs, populations, and confounder adjustments. Inherent biases and unmeasured confounding factors, though mitigated, remained a concern. The discrepancy between observational studies and RCTs may stem from the former’s inherent biases and the latter’s controlled environment, better capturing the intervention’s true effect. Moderate heterogeneity was detected among RCTs included in this meta-analysis, but unmeasured confounders appeared more impactful in the observational studies meta-analysis. Inconsistencies suggest that the increased risk observed in patients on PPI therapy could reflect their higher inherent risk due to poor prognosis, regardless of PPI use. In real-world practice, patients with high bleeding risk are more likely to receive proton pump inhibitors PPIs than those without high bleeding risk, and these patients also possess high ischemic risk. Therefore, patients who are prescribed PPIs may have high ischemic risks. This could be a reason for the discrepancy between the results of observational studies and RCTs.

Assessing the combined PPI effect should consider individual drug profiles rather than amalgamating data from multiple drugs in a class. Although we aimed to lessen the impact due to discrepancies in baseline characteristics among patients using PPIs and non-users through adjusted estimates from each study, some provided only unadjusted data.

Several prior meta-analyses have examined the link between PPI use and the risk of CVD. For instance, Jeridi et al.‘s analysis indicated that PPIs, as a category of drugs, did not correlate with a heightened risk of cardiovascular events [6]. Nonetheless, mixed outcomes emerged for concurrent use of PPIs with clopidogrel. Specifically, examining omeprazole’s impact on cardiovascular well-being suggested no adverse effects. Another meta-analysis from prospective observational studies implied that short-term PPI use for treating gastroesophageal conditions did not elevate the risk of initial cardiovascular incidents [9]. The reported increase in cardiovascular mortality has been largely attributed to publication bias and the inherent biases of observational studies, such as unmeasured confounding variables and indication bias. Considering these findings alongside RCT outcomes, it appears dubious to consider PPI consumption as an independent CVD risk factor, separate from any potential interaction with clopidogrel.

The clinical implications of this study are significant for the management of patients PCI. The divergent findings from observational studies and RCTs underscore the necessity for clinicians to critically evaluate the use of proton PPIs in the context of DAPT. Given that PPIs are widely prescribed to mitigate gastrointestinal bleeding risks in patients on antiplatelet therapy, understanding their potential cardiovascular impact is paramount. While observational studies suggest an increased risk of adverse cardiovascular events with PPI use, RCTs do not corroborate this finding. Therefore, it may not be necessary to universally avoid PPIs in patients post-PCI if they have a clear indication for their use, particularly in those with a high risk of gastrointestinal complications. Clinicians should consider the individual patient’s risk profile, the specific PPI being prescribed, and the potential for drug-drug interactions, particularly with clopidogrel.

Future research should focus on large-scale, prospective studies that can control for the wide array of confounding factors not accounted for in observational studies. Investigations should delineate the cardiovascular risks associated with individual PPIs, as there may be variability within this drug class. There is also a need for further pharmacodynamic studies to understand the interactions between PPIs and DAPT, especially with newer antiplatelet agents. Additionally, genomic studies may provide insights into the variability of patients’ responses to combined PPI and antiplatelet therapy.

The present study has a few limitations. The inclusion of studies was restricted to those published exclusively in English, potentially introducing language bias and omitting relevant data available in other languages. Most included studies adjusted for multiple confounders, but the adjustment variables varied significantly between studies, potentially leading to residual confounding. Also, the analysis depended heavily on the quality of the included studies, where the presence of unreported biases within the original studies could skew the meta-analysis results. The analysis of observational studies may be particularly susceptible to indication bias, as patients prescribed PPIs might have had higher baseline risks for cardiovascular events. The scope of PPIs reviewed was broad, and individual PPI effects, which may vary significantly in terms of cardiovascular risk, were not extensively differentiated, which could mask specific risks associated with particular PPIs. These limitations indicate the requirement for cautious interpretation of the findings and suggest a pressing need for more targeted research that can provide clearer insights into the cardiovascular safety of specific PPIs in the post-PCI setting. While PPIs are essential for gastrointestinal protection, especially post-PCI, their potential cardiovascular risks warrant a tailored approach. Further investigations should focus on individual PPIs and consider patient-specific factors to refine our understanding of the cardiovascular implications of PPI use. Furthermore, studies should focus on individual PPIs rather than combines effect of PPI class.

## Conclusion

The current evidence is insufficient to establish a definitive relationship between PPI use and adverse cardiovascular events in individuals undergoing PCI due to conflicting results between RCTs and observational studies. More comprehensive, high-quality studies are needed to clarify this relationship, particularly studies that can better control for confounding factors and provide a more nuanced analysis of the effects of individual PPIs.

### Electronic supplementary material

Below is the link to the electronic supplementary material.

Supplementary Material 1

## Data Availability

All data are presented within the manuscript and are available by contacting the corresponding author.
