# Supplementary material for: Adverse cardiovascular outcomes associated with proton pump inhibitor use after percutaneous coronary intervention: a systematic review and meta-analysis
Source: BMC Cardiovasc Disord. 2024 Jul 17;24:372. doi: 10.1186/s12872-024-04029-0 (PMC11253415; doi:10.1186/s12872-024-04029-0)
Supplement: Supplementary file 1 — Supplementary Material 1 [file 12872_2024_4029_MOESM1_ESM.docx]

**Supplementary Materials**

**Table S1.** PRISMA Checklist

**Table S2.** The adjusted search terms as per searched electronic databases

**Table S3.** Risk of bias assessment of studies

**Figure S1:** Funnel plot of Observational studies

**Figure S2:** Funnel plot of RCTs

## **Table S1.** PRISMA Checklist

| **Section and Topic** | **Item #** | **Checklist item (Prevalence of kidney diseases among the dengue patients: A systematic review and meta-analysis)** | **Location where item is reported** |
| --- | --- | --- | --- |
| **TITLE** | | |  |
| Title | 1 | Identify the report as a systematic review. | 1 |
| **ABSTRACT** | | |  |
| Abstract | 2 | See the PRISMA 2020 for Abstracts checklist. (made as per the Journal guidelines) | 2 |
| **INTRODUCTION** | | |  |
| Rationale | 3 | Describe the rationale for the review in the context of existing knowledge. | 3 |
| Objectives | 4 | Provide an explicit statement of the objective(s) or question(s) the review addresses. | 3 |
| **METHODS** | | |  |
| Eligibility criteria | 5 | Specify the inclusion and exclusion criteria for the review and how studies were grouped for the syntheses. | 4 |
| Information sources | 6 | Specify all databases, registers, websites, organisations, reference lists and other sources searched or consulted to identify studies. Specify the date when each source was last searched or consulted. | 4, Table S3 |
| Search strategy | 7 | Present the full search strategies for all databases, registers and websites, including any filters and limits used. | Table S3 |
| Selection process | 8 | Specify the methods used to decide whether a study met the inclusion criteria of the review, including how many reviewers screened each record and each report retrieved, whether they worked independently, and if applicable, details of automation tools used in the process. | 4 |
| Data collection process | 9 | Specify the methods used to collect data from reports, including how many reviewers collected data from each report, whether they worked independently, any processes for obtaining or confirming data from study investigators, and if applicable, details of automation tools used in the process. | 4 |
| Data items | 10a | List and define all outcomes for which data were sought. Specify whether all results that were compatible with each outcome domain in each study were sought (e.g., for all measures, time points, analyses), and if not, the methods used to decide which results to collect. | 3 |
|  | 10b | List and define all other variables for which data were sought (e.g., participant and intervention characteristics, funding sources). Describe any assumptions made about any missing or unclear information. | 4, Table S4 |
| Study risk of bias assessment | 11 | Specify the methods used to assess risk of bias in the included studies, including details of the tool(s) used, how many reviewers assessed each study and whether they worked independently, and if applicable, details of automation tools used in the process. | Table S4 |
| Effect measures | 12 | Specify for each outcome the effect measure(s) (e.g. risk ratio, mean difference) used in the synthesis or presentation of results. | 5 |
| Synthesis methods | 13a | Describe the processes used to decide which studies were eligible for each synthesis (e.g. tabulating the study intervention characteristics and comparing against the planned groups for each synthesis (item #5)). | 4,5 |
|  | 13b | Describe any methods required to prepare the data for presentation or synthesis, such as handling of missing summary statistics, or data conversions. | NA |
|  | 13c | Describe any methods used to tabulate or visually display results of individual studies and syntheses. |  |
|  | 13d | Describe any methods used to synthesize results and provide a rationale for the choice(s). If meta-analysis was performed, describe the model(s), method(s) to identify the presence and extent of statistical heterogeneity, and software package(s) used. | 4.5 |
|  | 13e | Describe any methods used to explore possible causes of heterogeneity among study results (e.g. subgroup analysis, meta-regression). | 5 |
|  | 13f | Describe any sensitivity analyses conducted to assess robustness of the synthesized results. | 6, Figure 3,4 |
| Reporting bias assessment | 14 | Describe any methods used to assess risk of bias due to missing results in a synthesis (arising from reporting biases). | 5 |
| Certainty assessment | 15 | Describe any methods used to assess certainty (or confidence) in the body of evidence for an outcome. | NA |
| **RESULTS** | | |  |
| Study selection | 16a | Describe the results of the search and selection process, from the number of records identified in the search to the number of studies included in the review, ideally using a flow diagram. | Table S2 |
|  | 16b | Cite studies that might appear to meet the inclusion criteria, but which were excluded, and explain why they were excluded. | NA |
| Study characteristics | 17 | Cite each included study and present its characteristics. | 4,5 Table 1 |
| Risk of bias in studies | 18 | Present assessments of risk of bias for each included study. | Table S4 |
| Results of individual studies | 19 | For all outcomes, present, for each study: (a) summary statistics for each group (where appropriate) and (b) an effect estimate and its precision (e.g. confidence/credible interval), ideally using structured tables or plots. | Table 1, Figure 2 |
| Results of syntheses | 20a | For each synthesis, briefly summarise the characteristics and risk of bias among contributing studies. | 4 |
|  | 20b | Present results of all statistical syntheses conducted. If meta-analysis was done, present for each the summary estimate and its precision (e.g. confidence/credible interval) and measures of statistical heterogeneity. If comparing groups, describe the direction of the effect. | 5,4 Figure 2 |
|  | 20c | Present results of all investigations of possible causes of heterogeneity among study results. | 5, Figure 3 |
|  | 20d | Present results of all sensitivity analyses conducted to assess the robustness of the synthesized results. | Figure 3 |
| Reporting biases | 21 | Present assessments of risk of bias due to missing results (arising from reporting biases) for each synthesis assessed. | NA |
| Certainty of evidence | 22 | Present assessments of certainty (or confidence) in the body of evidence for each outcome assessed. | NA |
| **DISCUSSION** | | |  |
| Discussion | 23a | Provide a general interpretation of the results in the context of other evidence. | 5,6, 7 |
|  | 23b | Discuss any limitations of the evidence included in the review. | 7 |
|  | 23c | Discuss any limitations of the review processes used. | 7 |
|  | 23d | Discuss implications of the results for practice, policy, and future research. | 7 |
| **OTHER INFORMATION** | | |  |
| Registration and protocol | 24a | Provide registration information for the review, including register name and registration number, or state that the review was not registered. | 3 |
|  | 24b | Indicate where the review protocol can be accessed, or state that a protocol was not prepared. | 3 |
|  | 24c | Describe and explain any amendments to information provided at registration or in the protocol. | NA |
| Support | 25 | Describe sources of financial or non-financial support for the review, and the role of the funders or sponsors in the review. | 8 |
| Competing interests | 26 | Declare any competing interests of review authors. | 7 |
| Availability of data, code and other materials | 27 | Report which of the following are publicly available and where they can be found: template data collection forms; data extracted from included studies; data used for all analyses; analytic code; any other materials used in the review. | Supplementary Materials |

**Table S3. The adjusted search terms as per searched electronic databases**

| Database | No | Search Query | Results |
| --- | --- | --- | --- |
|  | |  | |
| PubMed | **#1** | **((((((((("Proton pump inhibitor"[Title/Abstract]) OR ("Proton pump inhibitors"[Title/Abstract])) OR (Omeprazole[Title/Abstract])) OR (Esomeprazole[Title/Abstract])) OR (Lansoprazole[Title/Abstract])) OR (Pantoprazole[Title/Abstract])) OR (Rabeprazole[Title/Abstract])) OR (Dexlansoprazole[Title/Abstract])** Filters: **Free full text** | 10718 |
|  | **#2** | **(((((((((((((((((((("stroke"[Title/Abstract]) OR ("cardiovascular disease"[Title/Abstract])) OR ("coronary artery disease"[Title/Abstract])) OR ("heart failure"[Title/Abstract])) OR ("cardiovascular mortality"[Title/Abstract])) OR ("CVD"[Title/Abstract])) OR ("cardiac*"[Title/Abstract])) OR ("Arrhythmia"[Title/Abstract])) OR ("heart disease"[Title/Abstract])) OR ("myocardial*"[Title/Abstract])) OR ("congestive heart failure"[Title/Abstract])) OR ("ischemic heart disease"[Title/Abstract])) OR ("Aortic disease"[Title/Abstract])) OR ("Congenital heart disease"[Title/Abstract])) OR ("MI"[Title/Abstract])) OR ("Deep vein thrombosis"[Title/Abstract])) OR ("pulmonary embolism"[Title/Abstract])) OR ("Heart muscle disease"[Title/Abstract])) OR ("Pericardial disease"[Title/Abstract])** Filters: **Free full text** | 647736 |
|  | **#3** | **#1AND #2** | 576 |
| EMBASE | **#1** | **'proton pump inhibitor'**:ti,ab OR **'proton pump inhibitors'**:ti,ab OR **'omeprazole'**:ti,ab OR **'esomeprazole'**:ti,ab OR **'lansoprazole'**/exp OR **'lansoprazole'** OR **'pantoprazole'**:ti,ab OR **'rabeprazole'**:ti,ab OR **'dexlansoprazole'**:ti,ab | 54018 |
|  | **#2** | **'stroke'**:ti,ab OR **'cardiovascular disease'**:ti,ab OR **'coronary artery disease'**:ti,ab OR **'heart failure'**:ti,ab OR **'cardiovascular mortality'**:ti,ab OR **'cvd'**:ti,ab OR **'cardiac*'**:ti,ab OR **'arrhythmia'**:ti,ab OR **'heart disease'**:ti,ab OR **'myocardial*'**:ti,ab OR **'congestive heart failure'**:ti,ab OR **'ischemic heart disease'**:ti,ab OR **'aortic disease'**:ti,ab OR **'congenital heart disease'**:ti,ab OR **'mi'**:ti,ab OR **'deep vein thrombosis'**:ti,ab OR **'pulmonary embolism'**:ti,ab OR **'heart muscle disease'**:ti,ab OR **'pericardial disease'**:ti,ab | 2510769 |
|  | **#3** | #1 AND #2 | 3143 |
| WOS  advanced | **#1** | (((((((((((((((((((((((((((((((TI=("Proton pump inhibitor" )) OR TI=("Proton pump inhibitors" )) OR TI=("Omeprazole" )) OR TI=("Esomeprazole" )) OR TI=("Lansoprazole " )) OR TI=("Pantoprazole " )) OR TI=("Rabeprazole " )) OR TI=("Dexlansoprazole" )) OR (((AB=("Proton pump inhibitor" )) OR AB=("Proton pump inhibitors" )) OR AB=("Omeprazole" )) OR AB=("Esomeprazole" )) OR AB=("Lansoprazole " )) OR AB=("Pantoprazole " )) OR AB=("Rabeprazole " )) OR AB=("Dexlansoprazole" )))))))))))))))))))) | 29905 |
|  | **#2** | ((((((((((((((((((((((((((((((((((((((((TI=(stroke)) OR TI=(cardiovascular disease)) OR TI=(coronary artery disease)) OR TI=(heart failure)) OR TI=(cardiovascular mortality)) OR TI=(CVD)) OR TI=(cardiac*)) OR TI=(Arrhythmia)) OR TI=(heart disease)) OR TI=(myocardial*)) OR TI=(congestive heart failure)) OR TI=(ischemic heart disease)) OR TI=(Aortic disease)) OR TI=(Congenital heart disease)) OR TI=(MI)) OR TI=(Deep vein thrombosis)) OR TI=(pulmonary embolism)) OR TI=(Heart muscle disease)) OR TI=(Pericardial disease) OR ((AB=(stroke)) OR AB=(cardiovascular disease)) OR AB=(coronary artery disease)) OR AB=(heart failure)) OR AB=(cardiovascular mortality)) OR AB=(CVD)) OR AB=(cardiac*)) OR AB=(Arrhythmia)) OR AB=(heart disease)) OR AB=(myocardial*)) OR AB=(congestive heart failure)) OR AB=(ischemic heart disease)) OR AB=(Aortic disease)) OR AB=(Congenital heart disease)) OR AB=(MI)) OR AB=(Deep vein thrombosis)) OR AB=(pulmonary embolism)) OR AB=(Heart muscle disease)) OR AB=(Pericardial disease))))))) | 1894834 |
|  | **#3** | #1 AND #2 | 1256 |

**Table S3: Risk of bias assessment of studies**

**Cochrane RoB-2 for RCTs**

| Study | D1 | D2 | D3 | D4 | D5 | Overall |
| --- | --- | --- | --- | --- | --- | --- |
| Nicolau 2020 | High | Some concerns | High | High | Some concerns | High |
| Jensen 2017 | Some concerns | Low | High | High | Low | High |
| Ren 2011 | Some concerns | Low | High | High | Low | High |
| Wei 2016 | Low | Low | High | Some concerns | Low | High |
| Zhang 2015 | Low | High | High | High | Low | High |
| Yano 2012 | Some concerns | Low | High | Some concerns | Low | High |
| Zhang 2020 | Low | High | Low | Some concerns | Low | High |
| Gargiulo 2016 | High | Some concerns | High | High | Some concerns | High |

**Table S4: Newcastle-Ottawa scale (NOS) for Observational studies**

| **STUDY** | **SELECTION (max 4 points)** | | | | **COMPARABILITY (max 2 points)** | **OUTCOME (max 3 points)** | | | **SCORE (out of 9)** |
| --- | --- | --- | --- | --- | --- | --- | --- | --- | --- |
|  | Representativeness | Selection | Ascertainment | Demonstration of the outcome of interest was not present at start of study | Comparability the basis of the design or analysis | Assessment of outcome | Was follow-up long enough for outcomes to occur? | Adequacy of the follow-up |  |
| Aihara 2012 (1) | 1 | 1 | 1 | 1 | 2 | 1 | 1 | 0 | 8 |
| Burkard 2011(2) | 1 | 1 | 1 | 0 | 1 | 1 | 1 | 0 | 6 |
| Chandrasekhar 2016 (3) | 1 | 1 | 1 | 1 | 1 | 1 | 0 | 1 | 7 |
| Dunn 2013 (4) | 1 | 1 | 1 | 1 | 1 | 1 | 0 | 0 | 6 |
| Gargiulo 2016 (5) | 1 | 1 | 1 | 0 | 1 | 1 | 0 | 1 | 7 |
| Harjai 2011 (6) | 1 | 1 | 1 | 1 | 2 | 1 | 1 | 1 | 9 |
| Jensen 2017 (7) | 1 | 1 | 1 | 1 | 1 | 1 | 1 | 0 | 7 |
| Liu 2022 (8) | 1 | 1 | 1 | 1 | 2 | 1 | 1 | 0 | 8 |
| Macaione 2012 (9) | 1 | 1 | 1 | 1 | 1 | 1 | 1 | 0 | 7 |
| Maret-Ouda 2022 (10) | 1 | 1 | 1 | 0 | 1 | 1 | 1 | 0 | 6 |
| Nicolau 2020 (11) | 1 | 1 | 1 | 1 | 1 | 1 | 0 | 1 | 7 |
| Ono 2022 (12) | 1 | 1 | 1 | 1 | 1 | 1 | 1 | 1 | 8 |
| Ren 2011 (13) | 1 | 1 | 1 | 1 | 1 | 1 | 0 | 1 | 7 |
| Sarafoff 2010 (14) | 1 | 1 | 1 | 1 | 1 | 1 | 0 | 0 | 6 |
| Tentzeris 2010 (15) | 1 | 1 | 1 | 0 | 1 | 1 | 0 | 1 | 7 |
| Wei 2016 (16) | 1 | 1 | 1 | 1 | 2 | 1 | 1 | 1 | 8 |
| Weisz 2015 (17) | 1 | 1 | 1 | 1 | 1 | 1 | 1 | 0 | 7 |
| Yano 2012 (18) | 1 | 1 | 1 | 1 | 2 | 1 | 1 | 0 | 8 |
| Zhang 2020 (19) | 1 | 1 | 0 | 1 | 0 | 0 | 1 | 0 | 4 |
| Zhu 2017 (20) | 1 | 1 | 1 | 1 | 1 | 1 | 1 | 0 | 7 |
| Zou 2014 (21) | 1 | 1 | 1 | 1 | 2 | 1 | 1 | 0 | 8 |
| Aihara 2012 (1) | 1 | 1 | 1 | 1 | 1 | 1 | 1 | 0 | 7 |


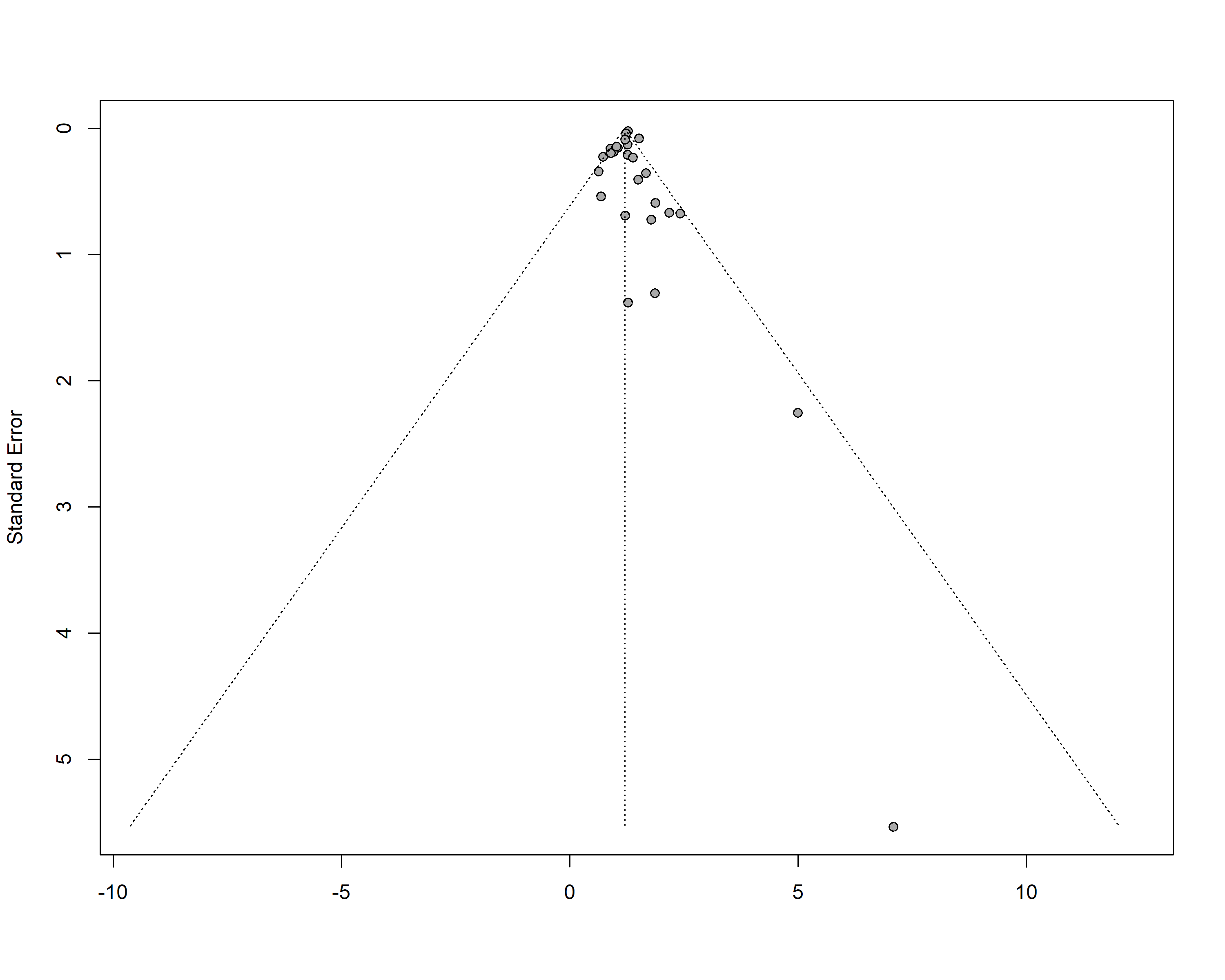


**Figure S1:** Funnel plot of observational studies


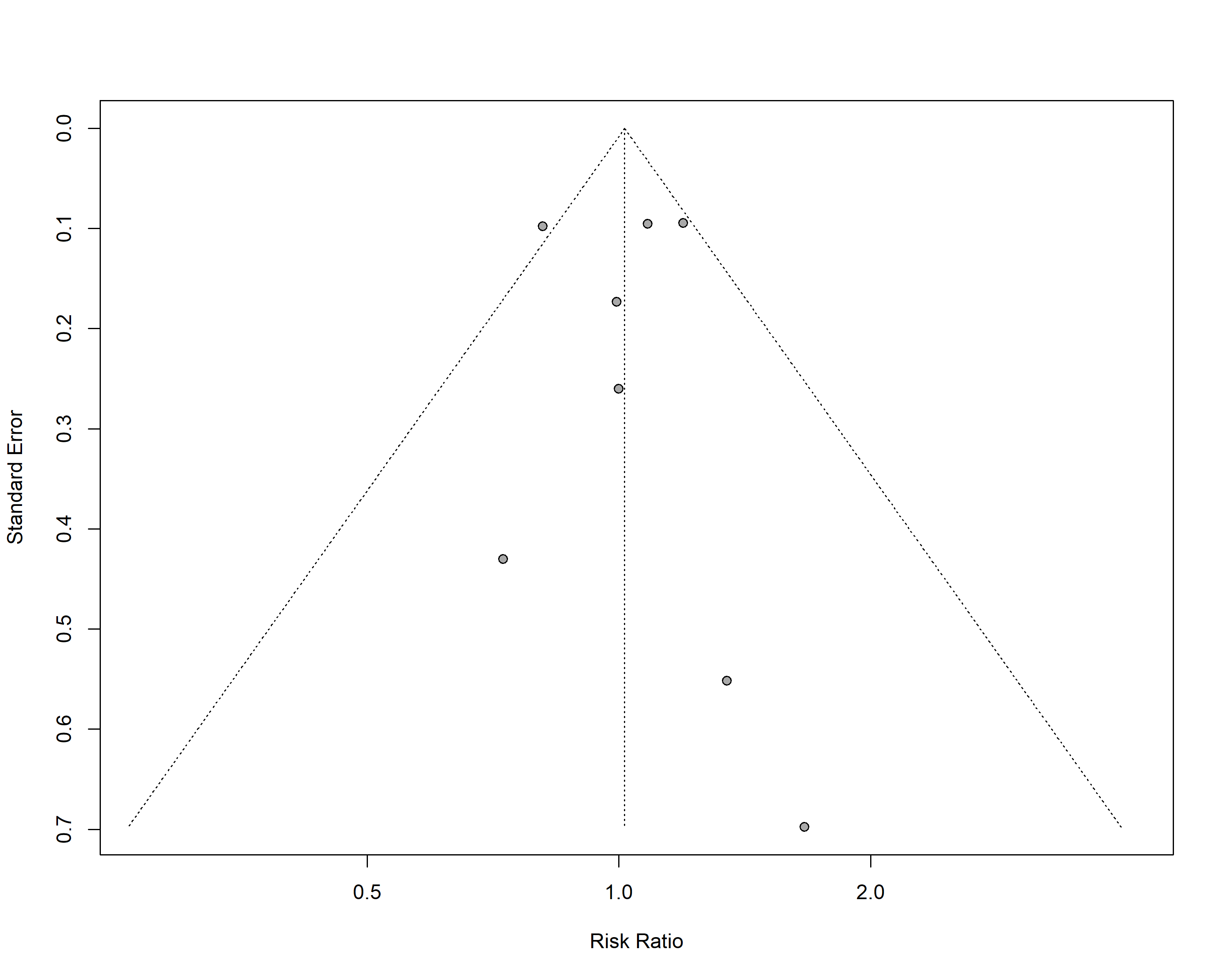


**Figure S2:** Funnel plot of RCTs

**Bibliography**

1. Aihara H, Sato A, Takeyasu N, Nishina H, Hoshi T, Akiyama D, et al. Effect of individual proton pump inhibitors on cardiovascular events in patients treated with clopidogrel following coronary stenting: results from the Ibaraki Cardiac Assessment Study Registry. Catheterization and Cardiovascular Interventions. 2012;80(4):556-63.

2. Burkard T, Kaiser C, Brunner‐La Rocca H, Osswald S, Pfisterer M, Jeger R, Investigators B. Combined clopidogrel and proton pump inhibitor therapy is associated with higher cardiovascular event rates after percutaneous coronary intervention: a report from the BASKET trial. Journal of internal medicine. 2012;271(3):257-63.

3. Chandrasekhar J, Bansilal S, Baber U, Sartori S, Aquino M, Farhan S, et al. Impact of proton pump inhibitors and dual antiplatelet therapy cessation on outcomes following percutaneous coronary intervention: Results From the PARIS Registry. Catheterization and Cardiovascular Interventions. 2017;89(7):E217-E25.

4. Dunn SP, Steinhubl SR, Bauer D, Charnigo RJ, Berger PB, Topol EJ. Impact of proton pump inhibitor therapy on the efficacy of clopidogrel in the CAPRIE and CREDO trials. Journal of the American Heart Association. 2013;2(1):e004564.

5. Gargiulo G, Costa F, Ariotti S, Biscaglia S, Campo G, Esposito G, et al. Impact of proton pump inhibitors on clinical outcomes in patients treated with a 6-or 24-month dual-antiplatelet therapy duration: Insights from the PROlonging Dual-antiplatelet treatment after Grading stent-induced Intimal hyperplasia studY trial. American heart journal. 2016;174:95-102.

6. Harjai KJ, Shenoy C, Orshaw P, Usmani S, Boura J, Mehta RH. Clinical outcomes in patients with the concomitant use of clopidogrel and proton pump inhibitors after percutaneous coronary intervention: an analysis from the Guthrie Health Off-Label Stent (GHOST) investigators. Circulation: Cardiovascular Interventions. 2011;4(2):162-70.

7. Jensen BE, Hansen JM, Larsen KS, Junker AB, Lassen JF, Jensen SE, De Muckadell OBS. Randomized clinical trial: the impact of gastrointestinal risk factor screening and prophylactic proton pump inhibitor therapy in patients receiving dual antiplatelet therapy. European Journal of Gastroenterology & Hepatology. 2017;29(10):1118-25.

8. Liu Y-H, Cao Z-Y, Dai Y-N, Zeng L-H, Zhang Y-S, Fan H-L, et al. Association of Proton Pump Inhibitor and Infection and Major Adverse Clinical Events in Patients With ST-Elevation Myocardial Infarction: A Propensity Score Matching Analysis. Frontiers in Medicine. 2022;9:882341.

9. Macaione F, Montaina C, Evola S, Novo G, Novo S. Impact of dual antiplatelet therapy with proton pump inhibitors on the outcome of patients with acute coronary syndrome undergoing drug-eluting stent implantation. International Scholarly Research Notices. 2012;2012.

10. Maret-Ouda J, Santoni G, Xie S, Rosengren A, Lagergren J. Proton pump inhibitor and clopidogrel use after percutaneous coronary intervention and risk of major cardiovascular events. Cardiovascular Drugs and Therapy. 2022:1-8.

11. Nicolau JC, Bhatt DL, Hohnloser SH, Kimura T, Lip GY, Miede C, et al. Dabigatran dual therapy vs warfarin triple therapy post-percutaneous coronary intervention in patients with atrial fibrillation with/without a proton pump inhibitor: a pre-specified analysis of the RE-DUAL PCI Trial. Drugs. 2020;80:995-1005.

12. Ono M, Onuma Y, Kawashima H, Hara H, Gao C, Wang R, et al. Impact of proton pump inhibitors on efficacy of antiplatelet strategies with ticagrelor or aspirin after percutaneous coronary intervention: Insights from the GLOBAL LEADERS trial. Catheterization and Cardiovascular Interventions. 2022;100(1):72-82.

13. Ren Y-h, Zhao M, Chen Y-d, Chen L, Liu H-b, Wang Y, et al. Omeprazole affects clopidogrel efficacy but not ischemic events in patients with acute coronary syndrome undergoing elective percutaneous coronary intervention. Chinese medical journal. 2011;124(06):856-61.

14. Sarafoff N, Sibbing D, Sonntag U, Ellert J, Schulz S, Byrne RA, et al. Risk of drug-eluting stent thrombosis in patients receiving proton pump inhibitors. Thrombosis and haemostasis. 2010;104(09):626-32.

15. Tentzeris I, Jarai R, Farhan S, Brozovic I, Smetana P, Geppert A, et al. Impact of concomitant treatment with proton pump inhibitors and clopidogrel on clinical outcome in patients after coronary stent implantation. Thrombosis and haemostasis. 2010;104(12):1211-8.

16. Wei P, Zhang Y-G, Ling L, Tao Z-Q, Ji L-Y, Bai J, et al. Effects of the short-term application of pantoprazole combined with aspirin and clopidogrel in the treatment of acute STEMI. Experimental and therapeutic medicine. 2016;12(5):2861-4.

17. Weisz G, Smilowitz NR, Kirtane AJ, Rinaldi MJ, Parvataneni R, Xu K, et al. Proton pump inhibitors, platelet reactivity, and cardiovascular outcomes after drug-eluting stents in clopidogrel-treated patients: the ADAPT-DES study. Circulation: Cardiovascular Interventions. 2015;8(10):e001952.

18. Yano H, Tsukahara K, Morita S, Endo T, Sugano T, Hibi K, et al. Influence of Omeprazole and Famotidine on the Antiplatelet Effects of Clopidogrel in Addition to Aspirin in Patients With Acute Coronary Syndromes–A Prospective, Randomized, Multicenter Study–. Circulation Journal. 2012;76(11):2673-80.

19. Zhang F, Su S, Hou Y, Zhao L, Wang Z, Liu F, et al. Effects (MACE and bleeding events) of ticagrelor combined with omeprazole on patients with acute myocardial infarction undergoing primary PCI. Hellenic Journal of Cardiology. 2020;61(5):306-10.

20. Zhu P, Gao Z, Tang X-F, Xu J-J, Zhang Y, Gao L-J, et al. Impact of proton-pump inhibitors on the pharmacodynamic effect and clinical outcomes in patients receiving dual antiplatelet therapy after percutaneous coronary intervention: a propensity score analysis. Chinese Medical Journal. 2017;130(24):2899-905.

21. Zou J-J, Chen S-L, Tan J, Lin L, Zhao Y-Y, Xu H-M, et al. Increased risk for developing major adverse cardiovascular events in stented Chinese patients treated with dual antiplatelet therapy after concomitant use of the proton pump inhibitor. PLoS One. 2014;9(1):e84985.
